# Patient advocate perspectives on involvement in HTA: an international snapshot

**DOI:** 10.1186/s40900-016-0052-9

**Published:** 2017-01-10

**Authors:** Anna Mae Scott, Janet L. Wale, On behalf of the HTAi Patient and Citizen Involvement in HTA Interest Group, Patient Involvement and Education Working Group

**Affiliations:** 1 Centre for Research in Evidence Based Practice, Bond University, Gold Coast, QLD 4229 Australia; 2 Patient and Citizen Involvement in HTA Interest Group, HTAi, 11A Lydia Street, Brunswick, VIC 3056 Australia; 3 Health Technology Assessment international (HTAi), 1200 10405 Jasper Avenue, Edmonton, AB T5J 3N4 Canada

**Keywords:** Patient involvement, Patient engagement, Patient and citizen involvement interest group, Health technology assessment, HTA, Survey

## Abstract

**Plain English summary:**

A number of health technology assessment (HTA) organisations have developed processes to engage patients in the assessment of new health technologies such as pharmaceuticals, diagnostic tests, devices or medical procedures. Typically, this involves the HTA agency providing an opportunity for patient advocates and their patient organisations (support groups for patients with a specific disease or condition) to provide submissions detailing experiences with the disease and the health technology that is being assessed. While some literature exists about how *HTA agencies* view the engagement of patients in the HTA process, it is not yet clear how the *patient advocates and patient organisations* themselves view this engagement. To answer this question, we surveyed the views of patient advocates who were members of patient organisations known to be engaged in the process of HTA or evidence-based practice. Snowballing – that is, passing on the survey invitation from individuals invited to take part in the survey to other individuals – occurred in one of the countries. The responses in this country provided a very useful comparison between the views of people who were appointed as the ‘patient representatives’ on an HTA committee with those who contributed input as part of the general patient organisation engagement process. Our findings identify gaps in understanding of the purpose of patient involvement and whether patient organisations felt their input made a difference, the information and support provided, and if and how feedback is given to the patient organisations. Our work can help inform further research as well as continuing improvements in HTA patient engagement processes.

**Abstract:**

**Background**

Patient involvement in health technology assessment (HTA) processes is becoming more frequent. However, it is not clear how patient advocates and their disease-based patient organisations that are involved in HTA view their involvement. We report on the results of an international survey of patient advocates and members of patient organisations about their experiences and perceptions of that involvement.

**Methods**

A 16-question survey was sent out to patient advocates and members of patient groups known to be involved in HTA processes or evidence-based practice. The survey consisted of open-ended questions focusing on respondent characteristics, stage and nature of involvement, support from HTA agencies for involvement, purpose of involvement, feedback on involvement, and whether the respondents felt that their input made a difference.

**Results**

Of 16 individuals who received the survey, 15 responded. Three, from Italy, Israel and Japan, were not involved in HTA in their country. Respondents from the following countries reported involvement in HTA processes: Canada, England, Scotland, and Wales, The Netherlands, Australia, Taiwan. The respondents indicated that HTA agencies reach out to them either actively or passively, and that their involvement is often at the appraisal stage of HTA. Typically, they reported involvement as either participants in committees or providers of submissions to HTA agencies. A wide range of approaches to supporting patient involvement by the HTA agencies was identified by respondents – including personal and telephone support, online resources, training and provision of information – but the level and type of support reported was uneven across jurisdictions. Not all respondents were clear on the purpose of their involvement in HTA, although some were able to cite specific examples of how their input made a difference; members of an HTA decision-making committee appeared to have a better understanding and were able to give examples. Feedback from HTA agencies to the patient groups on their submissions is often not provided.

**Conclusions**

Although considerable progress has been made in terms of engaging patients and patient groups in HTA, gaps remain in how involvement is supported, including facilitating involvement, clarity on roles, two-way flow of information, and methods for enhancing communication between patient organisations and HTA agencies.

## Background

### Health Technology Assessment (HTA)

Health technologies include pharmaceuticals, diagnostic tests, medical devices and procedures. Health technology assessment (HTA) is the “systematic evaluation of the properties and effects of a health technology, addressing the direct and intended effects of this technology, as well as its indirect and unintended consequences” [1]. This is accomplished by evaluating health technologies for their clinical effectiveness, cost-effectiveness, safety, social and economic characteristics [2, 3]. The aim of HTA is to make evidence-based decisions about public funding of health technologies e.g. by placing (or not placing) health technologies on national formularies.

### Patient and public involvement in HTA

Although historically, patient and public involvement (PPI) in HTA processes and decision-making has not been extensive, such involvement is growing internationally. Initiatives and projects aimed at facilitating such involvement have been observed in the United Kingdom, Europe, Canada, Asia and Australia [4–12].

Issues around patient and public involvement in HTA are often discussed under the single umbrella of ‘PPI’, however, a distinction needs to be drawn between patients and the public as their interests are not necessarily aligned and at times may even conflict [13]. ‘Patients’ can be construed as (actual or potential) users or beneficiaries of a health technology, who have experiential knowledge about a health condition to which the health technology applies, or the health technology itself. Carers also have personal experience of living with the condition and in some instances may speak on behalf of patients. The ‘public’, on the other hand, are those members of a wider community who have an interest in the well-being and sustainability of the health system. They have insights into social aspects of HTAs but lack personal experience with the health condition or the health technology itself [13–15]. The ‘public’ is a broad category that can include members of the general public (sometimes referred to as ‘citizens’) as well as organisations that represent the interests of consumers as users of health care, carers and patients themselves [12].

Two key sets of reasons for involving patients in HTA are generally cited in the literature. The first set of reasons focuses on transparency, legitimacy and fairness in decision making. Patient participation is viewed as a means of enhancing trust in the HTA decision-making process and acceptance of the resulting decisions. The second set of reasons centres around the patients’ evidentiary contributions. Patients are seen as contributors of valuable first-hand experiential knowledge of living with a particular health condition; they have experience with the health technology under assessment, or currently available technologies, the use of associated health services, and associated benefits, risks and side effects [4, 11, 13, 15–19]. This is where the distinction between patients and the public becomes acutely salient, as it is only the patients and possibly their carers (but not the general public) who have this personal knowledge and are able to provide this kind of information.

It is recognised that patient involvement can – and should – take place across the entire HTA process [14]. Indeed, there is evidence that HTA agencies are increasingly involving patients in all stages of HTA. A recent review of the roles of patients and the public in HTA processes internationally, identified examples of patient involvement in the following stages of HTA: identification of health technologies for assessment (e.g. Australia, New Zealand, The Netherlands), priority setting or selection of technologies for assessment (e.g. France, USA), conduct of the HTA itself (e.g. Canada, England, Wales), review of assessment results and generation of recommendations (e.g. The Netherlands, Germany, Canada), implementing the funding recommendations (e.g. England, France, USA), and dissemination of the decisions (e.g. Australia, Canada) ([13]; see also [16, 19]).

### The nature of the present contribution

Patient involvement in HTA is becoming both more frequent, in that it is taking place in more agencies or countries, and more pervasive in that it is taking place at many stages of HTA. However, due to its relative novelty, formal examinations of the process and impact of patient involvement in HTA are only just beginning to take place and to be made available in journals [4].

Examinations of the HTA agencies’ initiatives often investigate PPI initiatives as a single unit of analysis, failing to differentiate between patients and the public (e.g. [12, 19, 20]). The analyses frequently approach PPI from the perspective of HTA agencies (e.g. [4, 12, 19, 20]), or from a mix of perspectives, combining the perspectives of HTA agencies themselves as well as scholars, policy makers, patients, and user group members (e.g. [17, 21]) or from the public perspective (e.g. [22]).

To our knowledge, to date no-one has focused specifically on the issue of what patient involvement in HTA looks like from the perspective of *patient advocates and the members of disease-based patient organisations* (hereafter ‘patient organisations’) involved in the process. It is therefore our aim to begin to fill this gap, by reporting on the results of a survey of patient advocates and members of patient organisations known to be involved in the HTA process about their experiences and perceptions of their involvement in the HTA processes. In particular, the purpose of the present research was to identify issues faced by patient advocates when making submissions to an HTA agency, either as part of the patient support group or as individuals.

## Methods

The questionnaire was jointly designed by the authors, representing extensive experience in processes of HTA both as a consumer representative and committee member (JW), and as an academic experienced in preparing HTAs and a member of HTA committees and HTAi’s Ethics Interest Group (AMS). The authors also have broad experience through membership of the Patient and Citizen Involvement in HTA Interest Group, with one (JW) being the Group’s former Chair.

The questionnaire was informed both by the existing literature in this area and the professional experience of the authors. The included questions were selected to align with the study objectives – to query issues specifically applicable to patient advocates and disease-based patient organisations (as opposed to HTA agencies, the public, industry, or researchers involved in HTA). The emphasis was on use of simple language, as it was anticipated that the survey would be completed by an international audience.

The questionnaire asked 16 questions, focusing on respondent characteristics, stage and nature of involvement, support from HTA agencies for involvement, purpose of involvement, feedback on input given, and whether the respondents felt that their input made a difference. All of the questions were open-ended in order to avoid suggesting any specific responses and to give the respondents an opportunity to explain their answers in-depth and in their own words. Examples were provided in the questionnaire where appropriate in order to facilitate providing answers. A copy of the questionnaire is provided in the Appendix.

Purposive sampling was applied to select respondees who could provide detailed and relevant information. The questionnaire, together with an introductory letter, was sent by e-mail to the selected international patient advocates on 14 March 2016. The recipients were selected on the basis of their interest in joining the HTAi Patient and Citizen Involvement Patient Panel, having worked with or as members of the HTAi Patient and Citizen Involvement in HTA Interest Group, or as being part of the Cochrane Collaboration Consumer Network and likely to be involved in evidence-based health care and therefore HTA. The recipients were asked to complete the questionnaire by 4 April 2016. To maximise the response rate, if the recipient felt unable to respond to the questionnaire, either because of the timeframe or if there was someone in their organisation or a related organisation who would be more able to do so, they were asked to forward the e-mail to that person. The recipients were also sent up to three reminders about the questionnaire, where necessary, in order to maximise the response rate.

Unexpectedly, snowballing of responses occurred in one of the countries (Canada) – the survey invitation was passed on by the original recipient to fellow ‘patient representatives’ on an HTA appraisal committee. This has led to this country having a high proportion of the total responses. It did, however, provide a very useful comparison between the views of ‘patient representatives’ who were appointed in that capacity as members of an HTA committee, with those of patients who contributed input into HTAs through their disease-based patient organisations.

The responses were succinct and thematically summarised by one of the authors. The analysis was checked by the second author and fed back to the respondents. The respondents were advised that as no personal or private data was being collected, a formal ethics approval was not required. Nevertheless, ethical practices for survey research were followed in that respondents’ anonymity was ensured and sufficient information was provided about the survey and its authors, to enable respondents to make an informed decision about participating. Respondents were informed that a return of a completed questionnaire implied a free and informed consent to share the information publicly.

## Results

### Profile of the respondents

Of the 16 individuals who received the survey, 15 responded. Countries represented by the respondents include: Canada, England, Scotland, and Wales, The Netherlands, Australia, Taiwan, Japan, Italy and Israel (Table 1). Despite several attempts, the authors were unable to obtain a response from Germany.

**Table 1:** Profile of respondents to the questionnaire

Country	Number of respondents	Respondent type		HTA agencies the respondents are involved with
		Patient representative on HTA agency’s committee	Member of a patient group [that potentially submits to an HTA agency]	
United Kingdom^a^	2	--	2	The National Institute for Health and Care Excellence (NICE); Scottish Medicines Consortium (SMC); All Wales Medicine Strategy Group (AWMSG); Various (rare diseases focus)
The Netherlands	1	--	1	Zorginstituut Nederland (ZIN, The National Health Care Institute)
Canada^a^	6	3	3	Canadian Agency for Drugs and Technologies in Health (CADTH) Common Drug Review (CDR); Pan-Canadian Oncology Drug Review (pCODR), Canadian Agency for Drugs and Technologies in Health (CADTH)
Australia^a^	1	--	1	Pharmaceutical Benefits Advisory Committee (PBAC); Medical Services Advisory Committee (MSAC)
Taiwan	2	--	2	The National Health Insurance Administration (NHIA); Pharmaceutical Benefits and Reimbursement Scheme (PBRS)
Japan	1	--	1	None
Italy	1	--	1	None
Israel	1	--	1	None

^a^Some respondents indicated involvement with more than one agency

Respondents from three countries (Italy, Israel, Japan) stated that they do not contribute to HTA activities in their country. This does not mean, however, that there is no patient involvement in any HTA activities in these countries, for example at a hospital level. The remainder of the article focuses on the responses received from: Canada, England, Scotland, and Wales, The Netherlands, Australia, Taiwan.

### How are the patient organisations reached, when, and what is their role

The respondents identified two main approaches adopted by the HTA agencies to reach out to patients and/or patient organisations (Table 2). First, the agencies contact the patient organisations that are registered with them, seeking submissions when a relevant health technology is being assessed. The second approach consists of agencies posting requests for submissions on the agency’s website and waiting for responses from patients, where applicable, and patient organisations (or the public).

**Table 2:** How are the patient groups identified, in what role are they involved, and when (at what stage)

Country	How does the agency reach out to patient groups?	Stage of HTA at which the patient representatives or patient groups are involved?	How are the patient representatives or patient groups involved in the HTA?
Scotland (SMC)	• Patient group is registered with the HTA agency, and notified by the HTA agency	• Appraisal stage	• Provide submission to the agency • Participate in meetings of patient groups, clinicians, agency
England and Wales (NICE, AWMSG, rare diseases)	• Patient group is registered with the HTA agency, and notified by the HTA agency • At scoping stage, stakeholders are asked to recommend patients [or patient groups • Through disease registry, newsletters or social media	• Scoping stage workshop • Appraisal stage • Appeals of recommendations • Scientific advice stage	• Provide patient experts who participate in workshops or committee meetings • Provide submission to the agency
The Netherlands (ZIN)	• Patient group is registered with the HTA agency • HTA agency reaches out to umbrella groups	• Appraisal stage	• Provide submission to the agency • Provide a statement at public meetings
Canada (CADTH CDR and pCODR)	Patient representatives Patient groups • Patient group is registered with the HTA agency, and notified by the HTA agency HTA agency posts on its website a call for submissions by patient groups	• Unclear answer (when submission goes to committee)	• As members of an HTA committee • Provide comments on draft recommendations (patient groups) Provide submissions to the agency (patient groups)
Australia (PBAC, MSAC)	• No answer provided, learn from industry	• Appraisal stage	• Provide submission to the agency • Hearings
Taiwan (NHIA, PBRS)	• Users registered online • HTA agency posts on its website a call for submissions by patients, groups	• Unclear	• Patient representatives attend committee meetings • Provide short submission to the agency

As seen in Table 2, patient advocates reported patient organisation involvement at various stages of the HTA process. However, this was predominantly at the appraisal stage, when the accumulated scientific and economic evidence about a technology is formally assessed by an HTA committee and contextualised against social and ethical issues. Less often, patient organisation participation was sought at the scoping stage to outline how the technology is to be assessed, or at a consultation stage in response to an HTA committee’s recommendations.

The types of involvement varied, although provision of submissions predominated. Patient organisations in all of the countries represented by the patient advocates responding to our survey reported this type of involvement in HTA. Additionally, patient advocates reported involvement of their organisations through provision of comments on recommendations, provision of patient experts to the HTA agency, and participation in hearings or meetings.

### Support for ‘patient representatives’ and patient groups involved in HTA

‘Patient representatives’ (as designated by the HTA agency concerned) on an HTA committee reported receiving a wide range of support from HTA agencies. The support included: a dedicated staff person at the HTA agency, provision by the HTA agency of orientation, online resources, webinars, monthly teleconferences, and supported attendance at annual symposia. Patient advocates involved with disease-based patient organisations reported that some (although not all) HTA agencies dedicate a staff member as contact person or have a dedicated PPI team. Other approaches reported include: HTA agencies offering guidance or framework documents (either via e-mail or on the agency’s website), arranged meetings with patient organisations to provide guidance and information, and facilitating contact between patient organisations to enable peer support (Table 3).

**Table 3:** Support for patient representatives and patient groups involved in HTA

Country	What kind of support and guidance is offered by the agency?	What kind of training is offered by the agency?	What kind of information on the health technology is provided, and by whom?
Scotland (SMC)	• Guidance offered by agency’s Patient and Public Involvement team • Guidance documentation exists • A clear framework for submission of input is provided on the agency website • Individuals participating in the process are greeted and briefed • Peer support is provided – group can contact other patient groups with experience in process • Has Public Involvement Network Advisory Group	Training and capacity building, ad hoc or yearly	• A form (recently revised by patients together with pharmaceutical industry) with information is provided
England and Wales (NICE, AWMSG, rare diseases)	• Guidance offered by agency’s Public Involvement team • Contact person is provided • Guidance documentation exists and is sent out • Individuals participating in the process are greeted and briefed • A clear framework for submission of input is provided on the agency website • Peer support is provided – group can contact other patient groups with experience in submissions/process • Contact team exists but is not yet well established • No formal contact is provided • Support is provided for key group personnel	• Training and capacity building, ad hoc or yearly • Training is provided for patient groups but it is not specific for patient groups and not regular • No	• During scoping stage, background is provided on the drug • It is assumed we know about the treatment but we can request further information • It is possible to request information but no formal mechanism for this exists • The information is very limited – from clinical trials; if a patient has been in a relevant trial, the group obtains information from that patient
The Netherlands (ZIN)	• No support is provided • A guidance document exists but requires updating	• No	• The group utilises whatever information its patients or umbrella organisation has • The media
Canada (CADTH CDR and pCODR)	Patient representatives on HTA Committees • Orientation is provided • Topic orientated speakers • Online resources and sent materials • Monthly teleconference • Attendance at annual symposium Patient groups(provide input and/or feedback) • Online guidance and information • Agency has a contact person to answer questions • Has a patient advisory group (Patient Community Liaison Forum)	• Training in HTA • Training through group courses^a^ • Webinars are provided • No formal training; mentoring is done within the patient group itself	• Obtain information from the drug company • Obtain information from patients who have participated in clinical trials or are waiting for access • From conferences, journal articles
Australia (PBAC, MSAC)	• No clear guidelines	• No	• The group is informed by the industry • Search for clinical trial data on our own
Taiwan (NHIA, PBRS)	• Brief guidance provided on the agency’s platform • Meeting with patient groups to discuss involvement and provide information and guidance	• Training provided by International Research Based Pharmaceutical Manufacturers Association, Taiwan Association of Patient Organisations, • Training is provided by Taiwan Society for Pharmacoeconomics and Outcome Research	• Information is sought from pharmacists, doctors, patient groups and monthly symposia for patients • Information is sought from clinical trial staff in hospital, trial patients, patient organisations; HTA report; International organisations for relevant disease area, pharmaceutical companies

^a^information provided by ‘patient representatives’ sitting on an HTA agency’s committee, and members of the patient groups submitting to an HTA agency, is separated out for clarity

Formal training was not often provided by HTA agencies to patient organisations, although some patient advocates reported that webinars are provided. Others reported that training is provided by industry and other (e.g. academic) organisations; training also takes the form of mentoring amongst the members of the patient organisations themselves.

Patient organisations often do not receive information about the technology under assessment from the HTA agency itself but, rather, rely on a wide range of other sources for that information. Those sources include: publicly available data, industry, participants in clinical trials or clinical trial staff, other members of the patient organisation itself, the media, internet searches, pharmacists, doctors, other patient organisations, existing HTA reports, conferences or patient symposia. Where agencies do provide information, their approaches include: provision of background and key information using a pre-designed form for the industry sponsor to complete; or the agency enables patient organisations to request further information from the HTA agency if required.

### Purpose, outcomes and feedback on the involvement

The understanding of the purpose of patient involvement in HTA varied among the respondents. Some respondents reported that the purpose was unclear to them, or that it was only somewhat clear; one respondent felt that patient involvement was an exercise in ‘ticking a box’. However, others understood patient involvement as an opportunity to provide HTA agencies with information on the experience of living with a condition or using existing treatments and the treatment under assessment. The patients can provide information on the value and impact of the treatment from a patient perspective, to help agencies understand unmet needs, provide input more generally, or help to set the content of a health insurance package (Table 4).

**Table 4:** Purpose, outcomes and feedback on the involvement

Country	Is the purpose of patient involvement clear, and if so, can you say what it is?	Does the input you provide make a difference, and if so, can you provide an example?	Does the HTA agency provide any feedback on how the patient group information was used and incorporated into decisions?
Scotland (SMC)	• To ensure appraisal committees understand impact of new drugs on quality of life; human perspective; patient experience of condition and treatment needs	• Weighting and impact [of input] not clear. • Help to create the ‘whole picture’ together with the industry and clinician information • Contact with advocacy groups of 1.5 years has led to being listened to, arguments heard	• Group is advised of the decision but no feedback is provided • Final reports or documents reference key points from patients, carers
England and Wales (NICE, AWMSG, rare diseases)	• To ensure appraisal committees understand impact of new drugs on quality of life; human perspective; patient experience of condition and treatment needs • Learning still – unclear	• Weighting and impact [of input] not clear. • Worked with company, clinicians to provide patient access scheme to increase/show value • Contact with advocacy groups of 1.5 years has led to being listened to, arguments heard	• Group is advised of the decision but no feedback is provided • Final reports or documents reference key points from patients, carers • Feedback is provided from meetings but may not be able to share it with the rest of the patient group
The Netherlands (ZIN)	• Sets the content of the insurance package, like G-BAZIN	• None since the Pompe, Fabry diseases example	• No, communicated through industry
Canada (CADTH CDR and pCODR)	Patient representatives • It should be clear: the experience of living with [a disease], treatments • Where value lies from the patient’s perspective, experience Patient groups • Yes – to give input • To hear from patients, check a box Somewhat – to understand impact on patients, fill unmet need	• It does make a difference. One drug was funded based on submission documenting how life was improved on treatment; identified relevant subgroup negatively affected by [a disease] • Importance of single measures/associated symptoms on daily life; relevance of quality of life data from clinical trials; unmet need in a patient subgroup • It demonstrates the impact on daily life, careers, finances, vision of life of carers in relation to quality adjusted life years • In drafting final recommendations • In final recommendations Educate committee about disease, available drugs and use, intolerability	• Patient submissions are referenced in both interim and final recommendations (reports) on agency’s website • Groups know their feedback is considered if a detail is queried • No information on how it is used, its value • Not yet Yes (no further detail provided), feedback
Australia (PBAC)	• Unclear	• No example provided	• No
Taiwan (NHIA, PBRS)	• Providing input, collecting patients’ opinions	• No answer provided	• An e-mail is sent acknowledging the submission • Submission is listed in the meeting minutes • No direct feedback is provided

In response to the question whether their input made a difference, a mixed picture emerged. Some respondents did not provide an answer or felt that their input has not made a difference for some time. Others cited a variety of ways in which their input has made a difference to the HTA process. ‘Patient representatives’ on HTA committees were able to point to specific funding decisions, for example, a decision to fund a drug due to information on improvement in quality of life, and identification of sub-groups that were particularly negatively impacted by the disease. Responses from patient organisations were more general, and focused on the areas where they felt their input has made a difference, such as: contextualising the quality of life data from trials, illustrating unmet need, clarifying the impact on daily life of the disease or health technology, helping to educate HTA personnel about the disease or its treatment, and helping to create a fuller evidentiary picture by adding to industry and clinician evidence. Some patient advocates noted that either the impact or the weight of their submissions, vis-à-vis the other evidence considered, is unclear.

Feedback is often not formally provided by the HTA agencies, and one respondent noted that this makes it difficult to know the value of the submission. However, some respondents reported that it can be gleaned whether the patient organisation submission was utilised, for example, by examining the meeting minutes to see if the submission is listed, or examining the interim and final recommendations (whether they make reference to patient organisation submissions or are changed following comment by the patient organisation). Where an agency queries the patient organisation about the content of their submission, it can also be inferred that the submission is being considered by the HTA agency. One respondent noted that feedback was provided (that their submission had been received) but did not furnish any details.

## Discussion

A mixed picture thus emerges about patient involvement in HTA from the perspective of patient advocates involved in the process. Patient advocates and ‘patient representatives’ report that HTA agencies involve patient organisations either actively, by reaching out to groups known to them (e.g. those listed in the HTA agency’s database), or passively by posting requests for input on the agency’s website. The passive approach has the drawback of placing the onus on the patient organisation to monitor the agency’s website for HTAs relevant to its members, or to be informed by industry. The active approach – HTA agency reaching out to patient organisations – means that patient organisations know when their input is being sought, and they can then notify their members. The HTA agencies using the active approach are generally open to adding patient organisations to their databases, although some have criteria that need to be met. Communication between patient organisations involved in similar or overlapping disease areas would also be beneficial, as this could further increase patient participation in the HTA process. Additionally, agencies may consider proactive advertising, since patient organisations may not necessarily be representative of patient populations in all cases.

Approaches to supporting disease-based patient organisation involvement in the HTA process include both personal and electronic means. Personal support involves dedicating an HTA agency staff member or team, enabling in-person meetings of patient organisations with HTA agencies, or facilitating interactions between patient organisations. Online approaches include provision of guidance or other documents (either via e-mail or by posting them on the agency’s website). Our questionnaire did not query which of these approaches is preferable to the patient organisations. It is possible that the answer to this question would vary by jurisdiction, HTA agency, and patient organisation. It could also depend on the size, remit and capacity of both the HTA agency and the patient organisation. Therefore, clear communication between the agency and patient organisations is of crucial importance. Individual agencies and patient organisations could benefit from considering approaches used by other agencies.

Questions about training for patients and patient organisations revealed that patients participating in HTA committees as ‘patient representatives’ receive training from the HTA agency. However, such training may not be provided by HTA agencies to patient organisations. Patient organisations may therefore turn to industry or other organisations, or facilitate internal mentoring within their own groups. This is less than ideal, as HTA agencies are best positioned to advise patient organisations about agency-specific processes and informational needs. Extending the training that is provided to ‘patient representatives’ on HTA committees to patient organisations and others could be a worthwhile endeavour.

A similar point can be made about information provision by HTA agencies to patient organisations. Although patient advocates report a wide range of creative approaches to obtaining information about technologies under assessment, the HTA agency or the industry sponsor for the new technology is in the best position to provide this information to patient organisations. Issues arise around confidentiality of the data provided to the agency, and codes of conduct created by overarching pharmaceutical company bodies to protect patients from direct advertising. An approach adopted in Scotland – of providing patient organisations with standardised information obtained using a form that has been co-designed by industry and patients and is completed by industry – may be worthwhile for adopting in other jurisdictions. Beyond that, initiatives reported in the literature around public involvement in HTA may also be helpful in terms of patient involvement in HTA. These include: providing patients participating in HTA committees with a clear and detailed description of the role, and preparing introductory documents about HTA and HTA agency processes for the involvement of patients and patient organisations together with other background and guidance materials [10, 23].

Although respondents provided a range of answers about the purpose of patient involvement in HTA, it was somewhat surprising that some of the respondents – involved in disease-based patient organisation submissions to HTA agencies – report a lack of clarity about the purpose of such involvement. This may point to informational needs by patient organisations that go beyond the need for information about the specific health technologies. This could include: the need for information about HTA itself and its goals in that jurisdiction; the process and methods adopted by the specific agency; the types of information and evidence considered during the technology assessment; and the specific role that information provided by patient groups plays. The last of these needs clarification as several respondents noted that the weight or impact of the patient submissions, vis-à-vis other evidence considered in the HTA process, was unknown to them. Providing this information could, moreover, help to resolve the identified lack of clarity about whether the information provided by the patient organisations makes a difference to the HTA processes and outcomes. Unsurprisingly, ‘patient representatives’ participating on HTA committees are better equipped to cite specific examples where patient submissions made a difference as they are part of the decision-making processes and so have first-hand experience of the discussions that take place. The patient advocates working with patient organisations were less able to do so, and answered the question by focusing on the type of input they felt made a difference (e.g. on daily experience with the disease, impact on quality of life).

Patient advocates with patient organisations report that although they can sometimes identify whether their submission has been utilised, feedback on the submissions is generally not provided to them by the HTA agencies. This is problematic as one of the dominant types of patient organisation involvement in the HTA process takes the form of written submissions, which is a resource intense activity for patient organisations. Feedback could inform the value of using those resources and additionally lead to directed quality improvements in submissions. HTA agencies are best positioned to identify their own informational needs and how the information provided was used by them [4]. CADTH in Canada (Common Drug Review) does now provide feedback to the patient organisations that make submissions, and has a policy of making the submissions easily accessible on its website, where it is given permission to do so, so that other patient organisations can learn from them [24]. SMC in Scotland also gives examples of useful submissions on their website so that other groups can learn from them and their PPI team will review submissions that are in preparation [25].

The challenges around involvement in HTA that were identified by patient advocates and their disease-based patient organisations when responding to our questionnaire are not surprising. Many of these same challenges to PPI have been identified by HTA agencies themselves. According to HTA agencies, some of the challenges include: tensions between scientific healthcare evidence and social considerations when considering the patient perspective; practical issues around engaging patients; dissemination of information to patient organisations; inadequate time to conduct high-quality engagement activities; the potential for lengthening the process of HTA by involving patients; lack of expertise in qualitative research within HTA agencies; how to address the diversity of patient populations, and identifying participants who are truly ‘representative’; and absence of resources, both within HTA agencies and patient organisations, to carry out meaningful engagement [19]. The picture that emerges from the present questionnaire is that a wide range of approaches have been adopted by HTA agencies to address some of these challenges. It is therefore hoped that some of the approaches identified here can serve as examples of the way forward both for HTA agencies and disease-based patient organisations. This may be particularly relevant to HTA agencies that have yet to involve patients or are in the early stages of involvement.

### Limitations and strengths

The questionnaire’s strength lies in its very high response rate (94%), which is considerably higher than the rates typically reported in the surveys conducted in the field of HTA of from 18–90% [12, 19, 20, 26]. The high response rate is attributable to many of the respondents being known to one of the authors (JW), who personally reached out to the respondents. However, it needs to be noted that – being the first survey focusing on the views of patient organisations and patient advocates – the survey was considerably smaller than other surveys in HTA, which also limits its generalisability. Another strength lies in the profile of respondents, which allowed us to compare the perspectives of patient advocates working within disease-based patient organisations involved in the HTA process with the views of ‘patient representatives’ who are members of the HTA committee.

A limitation lies in the potential for selection bias in the recruited sample of participants as the survey specifically targeted people known to be involved in HTA in their own countries. This targeting was deliberate, however, as the present survey was intended to identify the views of those patient advocates and patient organisation members known to have experience with HTA processes; it was not an effort to assess the awareness of patient advocates around HTA activities in their own countries, which will become the main focus of a future, more broadly distributed questionnaire.

It is worth noting that because more than half of the responses were from Canada and UK (in aggregate), the views presented here are of some of the most developed patient involvement initiatives, in terms of both longevity and comprehensiveness, and may not be representative of the views of patients and disease-based patient organisations involved in HTA processes in other jurisdictions. Identifying approaches to patient involvement in jurisdictions with more developed patient involvement initiatives does, however, offer an opportunity to those jurisdictions with less developed initiatives to identify options that can be adapted and adopted in their countries.

Finally, a potential limitation lies in the open-ended nature of the questions used in the survey, which may have led to their incorrect interpretation by non-English as first language speakers. To address this, a summary of the responses was sent to the respondents for their verification. Some adjustment in the wording of the summary was made in response to arguments presented to us by the ‘patient representatives’ on an HTA appraisal committee. This was related to patient organisations being able to give suggestions on draft recommendations and see changes in wording, which the ‘patient representatives’ saw as feedback on the patient organisation submissions. Otherwise, we are confident that the potential for this problem was minimised.

## Conclusions

The findings of this questionnaire suggest a mixed picture. Some progress has been made in terms of engaging patients through their disease-based patient organisations in the HTA process, in a way that is sensitive and appropriate to patient organisation needs. However, important gaps remain. Therefore, the next steps could include a careful consideration of an optimal balance between enhancing the opportunities for patient organisations to make contributions to HTA and the informational and process needs of the HTA agencies. Among the specific issues in need of consideration, are:

- What are the best processes for facilitating the involvement of disease-based patient organisations in HTA;
- How should patient organisation involvement be operationalised, e.g. what role(s) should patients take on, what training do the patient organisations need to enable this;
- How to enhance communication between HTA agencies and patient organisations vis-à-vis the purpose of involvement, processes, and expectations;
- How best to support the involvement of patient organisations in HTA, what types of ongoing training and quality improvement, information provision, feedback processes, etc., should be implemented.

As suggested by the questionnaire, patient involvement processes are constantly evolving and some agencies have already taken steps towards addressing at least some of the identified issues. Patient organisations recognise and appreciate those efforts. It is hoped that the findings presented here will continue to spur efforts towards further developments by these and other HTA agencies. A re-survey is planned in the future, this time co-designed with the patient members and patient group representatives.

## Appendix

**Table 5:** Questionnaire

Information sought	Response	Any further comments
1. Your name		
2. Your patient group affiliation		
3. The country(ies) in which you mostly work		
4. The agency or organisation that your patient group provides patient input for		
5. At what stage(s) of the process is this input sought? e.g. at scoping or protocol stage, for a scientific report, when the submission goes to the decision making committee		
6. Who is the input from? e.g. patients, carers, patient experts, public/consumers		
7. How are they involved? e.g. committee member, consultations, providing information, templated submissions, hearings		
8. Is there a framework for the input and is clear guidance provided by the agency or organisation?		
9. Is training or capacity building provided – and if so, by whom?		
10. Is the purpose of providing input clear – what do you think it is?		
11. How does the agency or organisation identify which patients, advocates or patient groups to contact for input? e.g. database held by agency/organisation, in newsletters, call for submissions on website		
12. What support does the agency or organisation provide? – you may wish to give an example		
13. What information do you receive about the new medication, and who provides it?		
14. Is feedback provided on the input? e.g. how the patient group/organisation’s submission was used and how it informed decision making		
15. Does the agency or organisation have a ‘patient advisory group’ to assist it in its work? - if so please describe		
16. Any other comments – and can you provide us with an example of when you felt your input made a difference?		

## Abbreviations

CADTH: Canadian Agency for Drugs and Technologies in Health
CDR: Common Drug Review
HTA: Health technology assessment
HTAi: Health Technology Assessment International
NICE: The National Institute for Health and Care Excellence
PCIG: HTAi Patient and Citizen Involvement in HTA Interest Group
SMC: Scottish Medicines Consortium

## References

[1] HTAGlossary.net. Health Technology Assessment (HTA) 2016. Available from: http://htaglossary.net/health+technology+assessment+%28HTA%29. Accessed 2 Aug 2016.

[2] EUR-ASSESS Steering Committee. Health technology assessment. Int J Technol Assess Health Care. 2009;25(Supplement 1):10.

[3] Government of Australia. Review of Health Technology Assessment in Australia: December 2009. 2009.

[4] Berglas S, Jutai L, MacKean G, Weeks L (2016). Patients’ perspectives can be integrated in health technology assessments: an exploratory analysis of CADTH Common Drug Review. Research Involvement and Engagement..

[5] Cleemput I, Christiaens W, Kohn L, Leonard C, Daue F, Denis A. Acceptability and Perceived Benefits and Risks of Public and Patient Involvement in Health Care Policy: A Delphi Survey in Belgian Stakeholders. Value Health. 2015;18(4):477–83. 10.1016/j.jval.2014.12.015 26091602

[6] Staniszewska S, Brett J, Mockford C, Barber R. The GRIPP checklist: strengthening the quality of patient and public involvement reporting in research. Int J Technol Assess Health Care. 2011;27(4):391–9. 10.1017/S0266462311000481 22004782

[7] Wortley S, Wale J, Grainger D, Murphy P. Moving beyond the rhetoric of patient input in health technology assessment deliberations. Aust Health Rev. 2016. [E-pub ahead of print] 10.1071/AH15216 27224935

[8] Dipankui MT, Gagnon MP, Desmartis M, Legare F, Piron F, Gagnon J, et al. Evaluation of patient involvement in a health technology assessment. Int J Technol Assess Health Care. 2015;31(3):166–70. 10.1017/S0266462315000240 26062904

[9] Staniszewska S. Patient and public involvement in health services and health research: A brief overview of evidence, policy and activity. J Res Nurs. 2009;14(4):295–8.

[10] Oliver S, Milne R, Bradburn J, Buchanan P, Kerridge L, Walley T, et al. Involving consumers in a needs-led research programme: a pilot project. Health Expect. 2001;4(1):18–28. 10.1046/j.1369-6513.2001.00113.x PMC5060045 11286596

[11] Abelson J, Bombard Y, Gauvin FP, Simeonov D, Boesveld S. Assessing the impacts of citizen deliberations on the health technology process. Int J Technol Assess Health Care. 2013;29(3):282–9. 10.1017/S0266462313000299 23863188

[12] Hailey D, Werko S, Bakri R, Cameron A, Gohlen B, Myles S, et al. Involvement of consumers in health technology assessment activities by Inahta agencies. Int J Technol Assess Health Care. 2013;29(1):79–83. 10.1017/S026646231200075X 23217279

[13] Menon D, Stafinski T. Role of patient and public participation in health technology assessment and coverage decisions. Expert Rev Pharmacoecon Outcomes Res. 2011;11(1):75–89. 10.1586/erp.10.82 21351860

[14] Facey K, Boivin A, Gracia J, Hansen HP, Lo Scalzo A, Mossman J, et al. Patients’ perspectives in health technology assessment: a route to robust evidence and fair deliberation. Int J Technol Assess Health Care. 2010;26(3):334–40. 10.1017/S0266462310000395 20584364

[15] Facey KM (2011). Patient involvement in HTA: What added value?. Pharmaceuticals Policy and Law..

[16] Gagnon MP, Desmartis M, Lepage-Savary D, Gagnon J, St-Pierre M, Rhainds M, et al. Introducing patients’ and the public’s perspectives to health technology assessment: A systematic review of international experiences. Int J Technol Assess Health Care. 2011;27(1):31–42. 10.1017/S0266462310001315 21262085

[17] Gauvin FP, Abelson J, Giacomini M, Eyles J, Lavis JN. Moving cautiously: Public involvement and the health technology assessment community. Int J Technol Assess Health Care. 2011;27(1):43–9. 10.1017/S0266462310001200 21262071

[18] Rowland P, McMillan S, McGillicuddy P, Richards J. What is “the patient perspective” in patient engagement programs? Implicit logics and parallels to feminist theories. Health (London, England: 1997). 2016. 10.1177/1363459316644494 27130651

[19] Whitty JA. An international survey of the public engagement practices of health technology assessment organizations. Value Health. 2013;16(1):155–63. 10.1016/j.jval.2012.09.011 23337227

[20] Hailey D, Nordwall M. Survey on the involvement of consumers in health technology assessment programs. Int J Technol Assess Health Care. 2006;22(4):497–9. 10.1017/S0266462306051427 16984683

[21] Lopes E, Street J, Carter D, Merlin T. Involving patients in health technology funding decisions: stakeholder perspectives on processes used in Australia. Health Expect. 2016;19(2):331–44. 10.1111/hex.12356 PMC5055264 25703958

[22] Wortley S, Tong A, Howard K. Preferences for engagement in health technology assessment decision-making: a nominal group technique with members of the public. BMJ Open. 2016;6(2):e010265. 10.1136/bmjopen-2015-010265 PMC4746444 26832433

[23] Royle J, Oliver S. Consumer involvement in the health technology assessment program. Int J Technol Assess Health Care. 2004;20(4):493–7. 10.1017/s0266462304001412 15609801

[24] CADTH. Updates for Patient Groups 2016. Available from: https://www.cadth.ca/about-cadth/what-we-do/products-services/cdr/patient-input/updates-patient-groups. Accessed 30 Aug 2016.

[25] SMC. Patient Group Partners 2016. Available from: https://www.scottishmedicines.org.uk/Public_Involvement/Patient_group_partners. Accessed 30 Aug 2016.

[26] Olry de Labry Lima A, García Mochón L, Caro Martínez A, Martín Ruiz E, Espín Balbino J. Mapping capacity to conduct health technology assessment in Central, Eastern and South-Eastern Europe. Croat Med J. 2016;57(1):66–70. 10.3325/cmj.2016.57.66 PMC4800330 26935616

